# The Efficacy of Two Mycotoxin Detoxifications on Laying Performance, Antioxidant Capacity, and Liver Damage of Laying Hens Fed Diet Naturally Contaminated with Low-Level Mycotoxins

**DOI:** 10.3390/vetsci12060520

**Published:** 2025-05-26

**Authors:** Huimin Ma, Wentao Cheng, Usman Nazir, Chengfei Wang, Haiming Yang, Xiaoli Wan

**Affiliations:** 1 College of Animal Science and Technology, Yangzhou University, Yangzhou 225009, China; mhm9401@163.com (H.M.); 19352672638@163.com (W.C.); usmann539@gmail.com (U.N.); hmyang@yzu.edu.cn (H.Y.); 2 Jiangsu Aomai Bio-Technology Co., Ltd., Nanjing 211225, China; 19816334320@163.com

**Keywords:** low-level mycotoxin, laying hen, laying performance, antioxidant capacity, liver damage

## Abstract

This study was conducted to investigate the effects of two mycotoxin detoxifications on laying hens. A total of 360 70-week-old Hy-Line Brown laying hens were randomly divided into 1 of the 4 dietary treatment groups, with 6 replicates per group and 15 hens per replicate. The laying hens of the four treatments were fed with a basal diet (CON), a diet with naturally low-level mycotoxin contaminated corn (the levels of AFB_1_, ZEA, and DON in the corn of the CON group were 1.68 μg/kg, 42.75 μg/kg, and 585.40 μg/kg, respectively), replacing 73% of the corn in CON (MC), the MC diet with 1 g/kg modified silica-aluminate mycotoxin adsorbent (MA), and the MC diet with 1 g/kg mycotoxin degrading enzyme and bacteria complexes degradation agent (MD), respectively. Liver tissue and serum samples were extracted at the end of the trial to assess the antioxidant status and hepatic injury biomarkers. The experimental data were preliminarily interpreted in Excel and then analyzed by one-way ANOVA using SPSS, version 25 (SPSS Inc., Chicago, IL, USA). The results showed that compared to the CON, the MC group had decreased laying rate (*p* < 0.05), ferric reducing antioxidant potential in serum (*p* < 0.05), and reduced glutathione (GSH) content in the liver, and increased feed/egg ratio, serum alanine aminotransferase (ALT) activity, and malondialdehyde (MDA) content. Compared to the MC group, feed/egg ratio and serum ALT activity in the MA group and MD group decreased (*p* < 0.05), while serum GSH content and superoxide dismutase activity in the liver of the MA and MD groups increased. The liver glutathione peroxidase activity and the egg yolk percentage in the MA group decreased (*p* < 0.05) compared to the MC group. Histopathological alterations in liver tissues induced by mycotoxin included vacuolar degeneration, hepatocyte necrosis and disintegration, inflammatory cell infiltration, and enlarged hepatic sinuses. In short, both MA and MD were effective in mitigating the combined effects of low-level mycotoxins on laying hen performance, antioxidant capacity and liver damage.

## Simple Summary

Mycotoxin is one of the major contaminants in feed and feed raw materials. Long-term exposure to multiple low-level mycotoxins in feed can cause negative effects. The current study evaluated the effects of mycotoxin adsorbent and its degradation on laying hens. The results demonstrated that detoxification methods effectively counteracted the negative effects, including reduced laying performance, liver, and oxidative damage induced by the combination of low-level mycotoxins.

## 1. Introduction

Toxic secondary metabolites known as mycotoxins are produced as filamentous fungi grow and multiply. Serving as both food and feed crop, corn is susceptible to a range of mycotoxins, which leads to the accumulation of mycotoxins and threatens human and animal health. Aflatoxin B_1_ (AFB_1_), deoxynivalenol (DON), and zearalenone (ZEA) have high detection rates, and their contamination is particularly severe [1]. Preventing feed mold is an effective way to reduce the negative impacts of mold on livestock and poultry production. However, production is often affected by several factors, including humidity, high temperatures, and other environmental conditions. Strict management from planting to storage, processing, and shipping of feed raw materials is essential to prevent mold [2]. Despite the best efforts to rigorously control mold at every stage, mycotoxin detection rates in feed components remain high. The coexistence of multiple low-dose mycotoxins poses a significant challenge, and the cumulative toxicity of multiple low-level mycotoxins is a growing problem. Some studies have found that even when the levels of AFB_1_, ZEA, and DON alone in diets are below the recommended level, they can still reduce growth performance, laying hen performance, and severely affect reproductive system function and immune responses [3,4,5,6]. While the combined effects of two or more mycotoxins in diets negatively affect the liver antioxidant capacity of laying hens, even when the levels of DON, ZEA, and AFB_1_ were below then China’s recommended level [7].

Mycotoxin detoxification can be accomplished in a variety of ways, including chemical, biological, and physical detoxification. Common physical methods include heat treatment, grain processing, cleaning, removal, adsorbent adsorption, and irradiation. While heat treatment can reduce mycotoxin levels, it may compromise grain nutritional value and processing quality [8]. Currently, adsorbent addition is the most widely used physical detoxification method in feed production [9]. Chemical detoxification involves using oxidants, reductants, or alkaline conditions to modify mycotoxin structures, breaking down toxic groups and reducing their harm [10]. However, chemical methods may produce harmful by-products and degrade dietary nutrients. Biological methods utilize microorganisms or enzymes to degrade mycotoxins by altering their chemical structure, effectively reducing toxicity [11].

Nowadays, activated carbon, modified montmorillonite, yeast cell wall extracts, and other macromolecular adsorbents are the main components in adsorption detoxification [1]. Adsorbents can reduce the toxic effects of mycotoxins in the feed of livestock and poultry by absorbing the mycotoxins. Thus, preventing them from being digested and absorbed by the animals as they pass through the digestive tract. It ensures that they are excreted directly from the body without being released into the system. Natural sodium silicoaluminate materials, such as zeolite, montmorillonite, bentonite, diatomite, and kaolin, have been widely employed and researched due to their large specific surface area, ion adsorption capacity, and specific affinity for mycotoxins [12]. Although natural sodium silicoaluminate minerals are effective at absorbing and delivering nutrients, their adsorption capacity and efficiency are limited in practice. However, modification of these natural minerals can enhance their selective adsorption of mycotoxins [13].

The biological strategies include the use of microorganisms and enzymes. Enzymatic detoxification, which is highly effective, adaptable, specific, and low in toxicity, can be carried out under moderate conditions without causing nutritional loss or secondary contamination [11]. Enzymatic detoxification is widely used in this era. The biological enzymes decompose mycotoxin into nontoxic substances to achieve detoxification. Pure enzymes offer an attractive and viable alternative to the use of whole bacterial cells, especially in environments that are unfavorable for microorganism survival. Though the identification, characterization, and purification of mycotoxin-degrading enzymes can be time-consuming and inefficient. However, the cloning and heterologous expression of enzymes through genetic engineering techniques enable efficient production with reduced labor and financial costs [2].

The objective of this study was to investigate the effects of two kinds of mycotoxin detoxification (modified silica-aluminate mycotoxin adsorbent and mycotoxin-degrading enzyme and degrading bacteria complex degradation agent) on laying performance, antioxidant capacity, and liver damage of laying hens fed a low-level mycotoxin-contaminated diet.

## 2. Materials and Methods

### 2.1. Ethical Statement

All animal research procedures in the present study were approved by the animal care and welfare committee of Yangzhou University (Yangzhou, China) (Animal Protocol Number: 202303082) and followed the Regulations for the Administration of Affairs Concerning Experimental Animals of the People’s Republic of China.

### 2.2. Materials

Mycotoxin adsorbent was purchased from Jiangsu Aomai Biotechnology Co., Ltd. (Nanjing, China). The main components of which are modified aluminosilicate minerals and mannan oligosaccharides. The information on producing the adsorbent was outside the scope of the paper and cannot be disclosed in detail due to commercial sensitivity.

Mycotoxin degradation agent was purchased from Jiangsu Aomai Biotechnology Co., Ltd. (Nanjing, China). Aflatoxin B_1_ and deoxynivalenol are degraded by bacterial enzymes, specifically those produced by Bacillus subtilis (total viable count ≥ 5 × 10^9^ CFU/g), and zearalenone is degraded by zearalenone-degrading enzymes (enzyme activity ≥ 100 U/g). The material is defatted rice bran. The information on producing the degradation agent was outside the scope of the paper and cannot be disclosed in detail due to commercial sensitivity.

Naturally low-level mycotoxin contaminated corn: Before the start of the experiment, slightly naturally contaminated corn was collected; the AFB_1_, ZEA, and DON levels in the naturally mycotoxin contaminated corn were 26.37 μg/kg, 261.43 μg/kg, and 613.31 μg/kg, respectively. No other mycotoxins were detected. During the experiment, corn and diet were stored in dry, cool, and ventilated conditions.

### 2.3. Animals, Diet, and Experimental Design

A total of 360 healthy 70-week-old Hy-Line Brown laying hens, with a similar egg production rate (80.69% ± 0.72%), were randomly divided into 1 of the 4 dietary treatment groups, with 6 replicates per group and 15 hens per replicate. The hens were housed in a well-ventilated, enclosed poultry house equipped with tiered cages. Environmental conditions were maintained at 22–25 °C with approximately 60% relative humidity, and a 16 h daily photoperiod was provided through artificial lighting.

The laying hens of the four treatments were fed a basal diet (CON), a diet with naturally low-level mycotoxin contaminated corn replacing 73% of the corn in CON (MC), the MC diet with 1 g/kg modified silica-aluminate mycotoxin adsorbent (MA), and the MC diet with 1 g/kg mycotoxin degrading enzyme and bacteria complexes degradation agent (MD). The pre-feeding period was 1 week, and the experimental period was 9 weeks. According to the pre-feeding period and previous summary [14,15], the addition of mycotoxin adsorbent and mycotoxin degradation agent in the diet was 1 g/kg. The basal corn–soybean meal diet was formulated according to NRC (1994) [16] to meet the nutrient requirements of laying hens (Table 1).

### 2.4. Mycotoxin Content Detection

Corn and diet were collected every two weeks for the detection of mycotoxin concentration. As for sampling, the sample was weighed into a 100 mL centrifuge tube. To perform the extraction process, the sample was homogenized for 3 min. Then, pure acetonitrile was added for the liquid sample pretreatment. The mixture was normally filtrated, and an aliquot of 15 mL of filtration was purified. Then, an aliquot of 10 mL of clean-up elution was transferred into a test tube and dried by nitrogen gas at 50 °C. The residue was redissolved by a mixture of methanol and ammonium acetate. The tube was subsequently capped and shaken about by vortex briefly to mix the content of the tube. Finally, the solution was passed through a nylon filter and ready for injection. The concentrations of AFB_1_ and ZEA were, respectively, detected by liquid chromatography-tandem mass spectrometry, and the concentration of DON was detected by high-performance liquid chromatography with immunoaffinity column clean-up.

### 2.5. Laying Performance

To assess the performance of egg production, total egg weight, egg count, and broken egg were daily measured. Daily feed intake was recorded weekly. Hen-day egg production, average daily feed intake, and feed/egg ratio were calculated with the following formulas:

Egg production(%)=total number of eggs(g)/number of laying hens alive/total number of days in the period×100;Broken egg rate(%)=number of broken eggs/total number of eggs×100;Average egg weight(g)=total eggs weight(g)/total number of eggs;Average daily feed intake(g/day)=total weekly feed consumption(g)/number of hens/days;Feed/egg ratio(g/g)=total feed intake(g)/total egg weight(g)

### 2.6. Sample Collection

At the end of the trial, 30 eggs were randomly selected from each group (5 eggs per replicate) for egg quality analysis. Birds were chosen at random. (1 bird from each replication). Blood was collected from wing veins, and serum was separated by centrifugation at 4 °C and 3000 r/min for 10 min. The serum was stored at −80 °C for subsequent analyses. After blood collection, the hens were euthanized by the cervical dislocation method. The left liver was cut into small pieces, placed in a 2 mL centrifuge tube, and then snap-frozen in liquid nitrogen and stored at −80 °C for further analysis. The same part of the right liver (1 cm × 1 cm × 1 cm) was placed in 4% paraformaldehyde for preparation of pathological sections.

### 2.7. Egg Quality

The egg weight, albumen height, haugh unit, and egg yolk color were measured using an egg analyzer (EMT-7300, Robotmation Co., Ltd., Tokyo, Japan). Egg shape index = transverse diameter/longitudinal diameter; eggshell ratio (%) = eggshell weight×100/whole egg weight; Egg yolk ratio (%) = weight of egg yolk × 100/weight of whole egg; The shell strength was measured using a shell strength instrument (AC220, Orka Company, Shanghai, China); the shell thickness (remove the inner shell membrane) was measured at three locations (equator, blunt, and sharp ends), and the values recorded at the 3 locations were averaged. The shell thickness was measured using an eggshell thickness gauge (Guilin Measuring Tool Cutting Tool Co., Ltd., Guilin, China).

### 2.8. Antioxidant Capacity

The contents of protein (TP, cat. No. A045-4-1), malondialdehyde (MDA, cat. No. A003-1-1), reduced glutathione (GSH, cat. No. A006-1-1), and the ferric reducing antioxidant potential (FRAP, cat. No. A015-3-1), activities of catalase (CAT, cat. No. A007-1-1), total superoxide dismutase (T-SOD, cat. No. A001-1-1), and glutathione peroxidase (GSH-Px, cat. No. A005-1-1) were measured with commercial kits from Nanjing Jiancheng Institute of Biological Engineering (Nanjing, China) and calculated from protein concentrations.

### 2.9. Liver Damage

At the end of the experiment, the activities of aspartate aminotransferase (AST, cat. No. C010-2-1) and alanine aminotransferase (ALT, cat. No. C009-2-1) in serum were determined by colorimetric kits from Nanjing Jiancheng Institute of Biological Engineering (Nanjing, China).

The liver samples fixed in 4% buffered formaldehyde were dehydrated, paraffin-embedded, stained with hematoxylin and eosin (H&E), and observed under a light microscope (ML31, Guangzhou Mshot Photoelectric Technology Co., Ltd., Guangzhou, China).

### 2.10. Statistical Analysis

The experimental data were preliminarily interpreted in Excel and then analyzed by one-way ANOVA through SPSS, version 25 (SPSS Inc., Chicago, IL, USA). The Kolmogorov–Smirnov test was used to assess the normality for all quantitative variables. Tukey’s HSD test was used for multiple comparisons, and *p* < 0.05 was considered significant. Data were presented as the mean and ±SEM.

## 3. Results

### 3.1. Mycotoxin Content in Diet

Mycotoxin content in diet results is presented in Table 2. The contents of AFB1 and ZEA in the MC, MA, and MD groups were significantly higher than those in the CON group (*p* < 0.05).

### 3.2. Laying Performance

Compared to the CON group, a significant increase in feed/egg ratio and a decrease in laying rate were found in the MC group. Compared to the MC group, the feed/egg ratio in the MA and MD groups was significantly decreased (*p* < 0.05), as shown in Table 3.

### 3.3. Egg Quality

The egg yolk percentage in the MA group was significantly decreased as compared to the MC group (Table 4) (*p* < 0.05).

### 3.4. Serum Antioxidant

The serum FRAP in the MC group was significantly reduced compared to the CON group, and serum MDA content was increased. The MA and MD groups had higher GSH levels than the MC group (*p* < 0.05), as presented in Table 5.

### 3.5. Liver Antioxidant

Liver antioxidants were analyzed by adding mycotoxin detoxification to a naturally contaminated corn diet on the liver antioxidant index of laying hens. Compared to CON, the liver GSH level was lower in animals from the MC treatment. The T-SOD activity in the liver of the MA and MD groups was significantly increased compared to the MC group. Compared to the MC group, the liver GSH-Px activity in the MA group was higher (Table 6).

### 3.6. Liver Damage

The serum ALT activity in the MC group was higher than in the CON group (*p* < 0.05). Notably, compared with the MC group, ALT activity in the MA and MD groups significantly decreased, as shown in Table 7.

**Figure 1:**
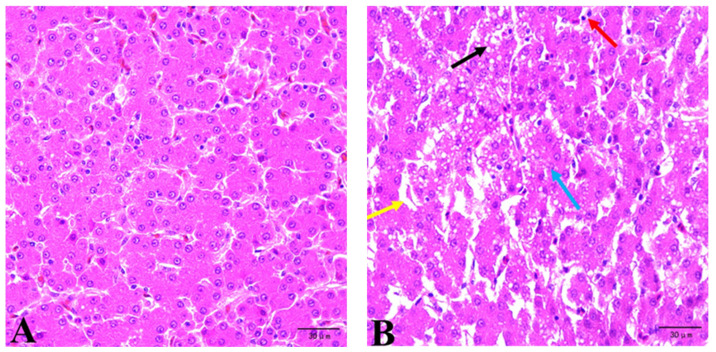

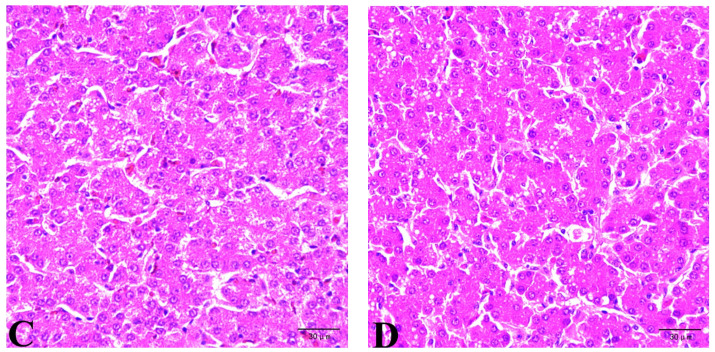
Photomicrographs of liver sections from different treatments. (**A**) basal diet (CON group), (**B**) naturally mycotoxin-contaminated corn substituting for 73.02% of corn in CON (MC group), (**C**) MC supplemented with 1 g/kg mycotoxin adsorbent (MA group), (**D**) MC supplemented with mycotoxin degradation agent (MD group), (H&E, 400×). Yellow arrow: widened hepatic sinuses; Black arrow: vacuolar degeneration; Bule arrow: hepatocyte necrosis and disintegration; Red arrow: inflammatory cell infiltration.

In the CON group, the liver showed normal hepatocyte structures and intact distinct nuclei without visible damage. No significant degeneration or pathological lesions were observed in the liver tissue (Figure 1A). The liver of birds in the MC group revealed severe lesions, including widened hepatic sinuses, different degrees of fatty vacuoles in hepatocytes, necrosis and disintegration of hepatocytes, and inflammatory cell infiltration (Figure 1B). The addition of mycotoxin detoxification to the mycotoxin-containing diet reduced the severity of lesions. Manifested as normal morphology and structure of hepatocytes, the decreased cytoplasmic vacuolation of hepatocytes, and the narrowed hepatic sinuses (Figure 1C,D).

## 4. Discussion

China’s latest Hygienical Standard for Feeds has established limit requirements for mycotoxins, including AFB_1_, ZEA, DON, and others. Similarly, the United States and the European Union have also set limit or provided guidelines for related mycotoxins. In this experiment, the levels of AFB_1_, ZEA, and DON in the diet were all below the mycotoxin recommended level of China’s “Hygienical Standard for Feeds” [17], the FDA of the USA [18], and the EFCA of the EU [19]. The levels of AFB_1_, ZEA, and DON in the corn of the CON group were 1.68 μg/kg, 42.75 μg/kg, and 585.40 μg/kg, respectively. Despite strictly testing the mycotoxin levels in the experiment, trace amounts of mycotoxins were still present in the corn and diet of the control group, suggesting that completely avoiding multiple low levels of mycotoxins in feed and feed ingredients during production is challenging.

Currently, most studies on mycotoxins focus on individual toxins, with limited attention given to the combined effects of mycotoxins. Multiple mycotoxin contaminations are common in feed materials, and exposure to a combination of toxins is more likely to have adverse health effects compared to a single toxin [20]. Zhu et al. [7] discovered that feeding moldy corn to laying hens decreased average egg weight and laying rate while increasing the broken egg rate. This study showed that feeding moldy corn significantly decreased the laying rate and increased the feed/egg ratio of laying hens. These effects may be attributed to the long-term accumulation of low doses of mycotoxins in the bodies of laying hens, leading to reduced feed intake and lower laying performance. The primary standards for evaluating the quality of eggs are their internal and external characteristics. External quality is determined by factors such as egg weight, egg shape index, eggshell thickness, and eggshell strength. While yolk color, albumen height, and Haugh unit are indicators used to assess the internal quality of the egg [21]. Mycotoxins can impair Ca^2+^ adsorption and transport and thus reduce egg quality. Studies have shown that treatment with DON, AFB_1_, and ZEA, either individually or in combination, resulted in decreased eggshell thickness, eggshell strength, and yolk color [22]. Additionally, Zhao et al. [6] found that mycotoxins also decreased the eggshell strength compared to the control group. However, the results of the present study were inconsistent with previous findings, which may be due to differences in poultry species, age, duration of exposure, and mycotoxin dosage.

The liver, as a major metabolic site and an important organ for material accumulation and detoxification, is the primary target organ of mycotoxins. One of the most important consequences of lipid peroxidation is the production of MDA, which is often used as an indicator of the redox state of tissues and cells. In general, lower MDA levels are negatively correlated with reactive oxygen species levels and are associated with improved cellular antioxidant capacity. Under normal conditions, the generation and removal of free radicals are balanced. However, aging and stress can increase free radical production, leading to oxidative damage and elevated MDA levels. Animals can produce endogenous antioxidant enzymes such as GSH-Px, SOD, and others to preserve the body’s dynamic equilibrium of free radicals [23]. Antioxidant enzymes, including SOD, CAT, GSH, and GSH-Px, are the first line of defense against the accumulation of oxidizing free radicals [24]. To mitigate oxidative damage, SOD catalyzes the conversion of superoxide free radicals into hydrogen peroxide, which is then broken down into water and oxygen molecules by GSH-Px and CAT; the combined effect of multiple mycotoxins on antioxidant activity in animals can lead to more severe oxidative stress [25]. Zhu et al. [7] found that the combined effect of moldy corn (containing 25.36 μg/kg AFB_1_, 245.05 μg/kg ZEA, and 990 μg/kg DON) significantly decreased the levels of T-AOC, T-SOD, and GSH in the liver while increasing the MDA content. This suggested that the combined exposure to multiple toxins weakens the capacity of eliminating free radicals. Histopathological examination serves as an effective method to assess the toxic effects of mycotoxins. Qiu et al. [26] observed the disorganized hepatic cord, mild blistering degeneration of hepatocytes, obvious perivascular mononuclear cell infiltration, and other pathological changes under the condition of low-concentration (10 μg/kg) AFB_1_ exposure. The ingestion of 12 µg/kg body weight (BW) DON and 40 µg/kg BW ZEA induced an increase in the liver lesion score, with the most frequent lesions being inflammatory infiltrate, congestion, cytoplasmic hepatocellular vacuolation, megalocytosis, trabecular disorganization, and necrosis [27]. Our study demonstrated that a diet with naturally low levels of mycotoxin contamination could increase serum MDA content and decrease liver GSH and serum FRAP content compared to the CON group. Histopathological results revealed liver morphological lesions in laying hens from the MD group. These results were consistent with previous research, which showed that animals fed diets contaminated with AFB_1_, DON, and ZEA, either individually or in combination, may experience liver damage, negatively affecting production and health status [13,28,29,30]. The increased MDA content and decreased GSH and FRAP levels indicate a disruption in the redox balance. Excessive free radicals attack hepatocytes, leading to hepatic vacuolation, infiltrative necrosis, and inflammatory cell disintegration. Additionally, serum ALT and AST levels are markers of improvements in cell viability and membrane penetration brought on by hepatic disturbance [31]. Serum biochemistry results indicated that mycotoxins increased ALT activity. Elevated ALT levels in the blood suggested liver tissue damage and injury, as observed in the present study.

The most common approach to reduce mycotoxin exposure involves decreasing their bioavailability through the use of mycotoxin-binding agents or adsorbents, which reduce mycotoxin absorption and subsequent distribution to the blood and the target organs. Based on their structural characteristics, aluminosilicates are classified into subcategories. The phyllosilicate family is one such group, distinguished by its sheet-like structure. Natural sodium silicoaluminate minerals can adsorb mycotoxins in a non-selective manner that may interact with the absorption of other essential nutrients [14]. In an in vitro gastrointestinal model simulation has been shown bentonite and montmorillonite can also adsorb protein [32,33]. Barrientos-Velázquez et al. [33] reported that bentonite adsorbed vitamin B_1_, indicating competition for adsorption sites between mycotoxins and nutrients. However, some studies have found that the addition of mycotoxin adsorbent does not affect the bioavailability of vitamins in vivo [34,35]. In our study, the inclusion of the mycotoxin absorbent appeared to mitigate adverse effects to some extent, leading to an increase in egg production rate, improved feed/egg ratio, and restored overall laying performance. This is attributed to the specificity of the mycotoxin adsorbent, which utilizes patented surface modification technology to selectively bind mycotoxins without adsorbing other nutrients. As a result, it efficiently adsorbs AFB_1_, ZEA, and DON, ensuring that the nutritional value of the diet meets the requirements for animal growth and production. However, the addition of mycotoxin adsorbent was observed to decrease the egg yolk ratio. Jahanian and Ashnagar [36] found that the addition of mannan-oligosaccharides decreases the concentrations of triglycerides and lowers levels of cholesterol. These findings suggest that mannan-oligosaccharides may influence lipid metabolism and fat deposition in egg yolks by reducing the availability of precursors required for yolk synthesis, leading to decreased yolk deposition and lower yolk weight. The precise mechanism underlying this effect requires further investigation in subsequent studies. Several studies have demonstrated that dietary adsorbents can enhance egg yolk weight, eggshell percentage, and eggshell thickness; improve eggshell strength and yolk color; and reduce mycotoxin-induced damage to egg quality [21,37]. According to the research, the addition of aluminosilicate minerals improved the antioxidant capacity of broilers by decreasing lipid peroxidation, increasing the activities of T-SOD and CAT, and decreasing the serum MDA levels [38]. In our study, the addition of mycotoxin adsorbent increased serum GSH levels and the activities of liver T-SOD and GSH-Px, indicating that mycotoxin detoxification alleviates oxidative stress and enhances the antioxidant performance of laying hens. Interestingly, dietary supplementation with a mycotoxin absorbent and degradation agent alleviated mycotoxin-induced histopathological alteration in the liver of laying hens. Furthermore, the dietary supplementation of mycotoxin absorbent mitigated the changes in ALT activity induced by mycotoxins. The findings were consistent with other studies demonstrating the ability of bentonite and yeast cell wall binders, smectite-based mycotoxin binders, mixed-dimensional attapulgite clay, and bentonite clay to reduce the detrimental effects of mycotoxins on animal biochemical parameters [21,30,39,40].

Enzymatic or microbial detoxification, commonly referred to as “biotransformation” or “biodetoxification”, involves the use of microorganisms or their purified enzymes to catabolize mycotoxins entirely or to transform them into less toxic compounds through modification or cleavage [41]. In the field of mycotoxin detoxification, biological approaches have garnered increasing interest and have become a prominent research hotspot. Mycotoxin degradation is primarily mediated by microbial strains that produce enzymes capable of converting toxins into less harmful or non-toxic metabolites. Numerous factors affect the degradation process, including the medium, the type of microorganism, the pH, bacterial cell concentration, and the incubation duration [42]. Enzymes offer the advantage of controlling the types of metabolites produced and can eliminate mycotoxins in a precise and well-defined manner compared to many other methods. Similar to mycotoxin adsorbent, mycotoxin degradation agents could effectively mitigate the adverse effects of mycotoxins on production performance, egg quality, antioxidant capacity, and liver damage.

## 5. Conclusions

In general, dietary supplementation with either a modified silica-aluminate mycotoxin adsorbent or a mycotoxin-degrading enzyme and bacteria complex effectively mitigates adverse effects induced by the combination of multiple low-level mycotoxins, including impaired laying performance of laying hens, decreased antioxidant capacity, and damaged morphological structure of liver tissue. These results demonstrate that mycotoxin detoxification can protect hens’ health and productivity and guarantee the safety of laying hens.

## Figures and Tables

**Table 1:** The composition and nutrient levels of basal diets (air-dried basis).

Items	Content (%)
Ingredients	
Corn	63.00
Soybean meal	25.00
Limestone	10.38
CaHPO_4_	0.80
Salt	0.30
DL-methionine	0.15
Premix ^1^	0.37
Total	100.00
Nutrient levels ^2^	
ME(MJ/kg)	10.99
CP	16.23
Ca	4.00
Available P	0.34
Methionine	0.40
Lysine	0.82
Threonine	0.61
Tryptophan	0.18

^1^ The premix provided per kilogram of diet: vitamin A, 10,000 IU; vitamin D_3_, 3000 IU; vitamin E, 15 IU; vitamin K_3_, 1.5 IU; vitamin B_1_ 1.0 mg; vitamin B_2_ 4.5 mg; vitamin B_6_ 3.0 mg; vitamin B_12_ 0.015 mg; nicotinamide, 25 mg; choline chloride, 300 mg; calcium pantothenate, 4.5 mg; folic acid, 0.85 mg; Fe (from ferrous sulfate), 70 mg; Cu (from copper sulfate), 8.0 mg; Mn (from manganese sulfate), 75 mg; Zn (from zinc sulfate), 80 mg; I (from calcium iodate), 0.6 mg; Se (from sodium selenite), 0.3 mg. ^2^ Nutrient levels are calculated values.

**Table 2:** Detection value of mycotoxin contents in diet.

Items ^1^	CON	MC	MA	MD	SEM	*p* Value
AFB_1_, μg/kg	1.28 ^c^	11.68 ^a^	8.73 ^b^	8.65 ^b^	1.020	<0.001
ZEA, μg/kg	58.12 ^b^	193.78 ^a^	162.38 ^a^	193.09 ^a^	14.203	<0.001
DON, μg/kg	426.03	506.13	483.96	452.68	14.893	0.252

^1^ AFB_1_: aflatoxin B_1_; DON: deoxynivalenol; ZEA: zearalenone. CON: basal diet; MC: naturally mycotoxin-contaminated corn substituting for 73% of corn in CON; MA: MC with 1 g/kg mycotoxin adsorbent; MD: MC with 1 g/kg mycotoxin degradation agent; SEM: standard error of the mean. ^a,b,c^ Values in the same row with different superscripts were significantly different (*p* < 0.05).

**Table 3:** The efficacy of two mycotoxin detoxifications on laying performance of laying hens fed diet naturally contaminated with low-level mycotoxins.

Item ^1^	CON	MC	MA	MD	SEM	*p* Value
laying rate, %	80.22 ^a^	74.73 ^b^	77.58 ^ab^	77.21 ^ab^	0.599	0.008
Broken egg rate, %	0.88	1.42	1.04	0.90	0.449	0.176
Average egg weight, g	63.21	63.75	63.94	63.89	0.182	0.496
Average daily feed intake, g	110.02	111.79	110.94	111.74	0.356	0.279
Feed/egg ratio, g/g	2.20 ^b^	2.35 ^a^	2.24 ^b^	2.24 ^b^	0.017	0.006

^1^ CON: basal diet; MC: naturally mycotoxin-contaminated corn substituting for 73% of corn in CON; MA: MC with 1 g/kg mycotoxin adsorbent; MD: MC with 1 g/kg mycotoxin degradation agent; SEM: standard error of the mean. ^a,b^ Values in the same row with different superscripts were significantly different (*p* < 0.05).

**Table 4:** The efficacy of two mycotoxin detoxifications on egg quality of laying hens fed diet naturally contaminated with low-level mycotoxins.

Items ^1^	CON	MC	MA	MD	SEM	*p* Value
Eggshell strength, kg/cm^2^	3.62	3.37	3.51	3.71	0.087	0.580
Eggshell thickness, mm	0.36	0.36	0.36	0.37	0.003	0.504
Eggshell ratio, %	10.29	10.53	10.77	10.75	0.083	0.132
Egg yolk ratio, %	26.15 ^a^	25.77 ^a^	24.01 ^b^	26.11 ^a^	0.298	0.027
Egg yolk color	7.17	7.20	7.10	7.03	0.112	0.965
Haugh unit	74.52	72.44	71.71	72.43	0.807	0.586
Egg shape index	1.32	1.32	1.31	1.31	0.003	0.504
Albumen height, mm	6.00	5.58	5.83	5.70	0.098	0.483

^1^ CON: basal diet; MC: naturally mycotoxin-contaminated corn substituting for 73% of corn in CON; MA: MC with 1 g/kg mycotoxin adsorbent; MD: MC with 1 g/kg mycotoxin degradation agent; SEM: standard error of the mean. ^a,b^ Values in the same row with different superscripts were significantly different (*p* < 0.05).

**Table 5:** The efficacy of two mycotoxin detoxifications on serum antioxidant index of laying hens fed diet naturally contaminated with low-level mycotoxins.

Items ^1^	CON	MC	MA	MD	SEM	*p* Value
FRAP, U/mL	18.77 ^a^	11.00 ^b^	11.59 ^b^	11.68 ^b^	0.799	<0.001
CAT, U/mL	3.97	2.78	3.07	3.27	0.298	0.600
T-SOD, U/mL	130.55	125.07	130.36	127.11	0.988	0.157
GSH, mg/mL	85.27 ^bc^	75.97 ^c^	91.47 ^ab^	98.02 ^a^	2.484	0.002
MDA, nmol/mL	27.69 ^b^	36.71 ^a^	30.90 ^ab^	31.15 ^ab^	1.086	0.027

^1^ CON: basal diet; MC: naturally mycotoxin-contaminated corn substituting for 73% of corn in CON; MA: MC with 1 g/kg mycotoxin adsorbent; MD: MC with 1 g/kg mycotoxin degradation agent. MDA: malondialdehyde; GSH: reduced glutathione; FRAR: ferric reducing antioxidant potential; CAT: catalase; T-SOD: superoxide dismutase; SEM: standard error of the mean. ^a,b,c^ Values in the same row with different superscripts were significantly different (*p* < 0.05).

**Table 6:** The efficacy of two mycotoxin detoxifications on liver antioxidant index of laying hens fed diet naturally contaminated with low-level mycotoxins.

Items ^1^	CON	MC	MA	MD	SEM	*p* Value
FRAP, U/mgprot	3.84	4.42	3.99	4.16	0.118	0.395
CAT, U/mgprot	72.62	70.66	72.87	75.10	1.157	0.623
T-SOD, U/mgprot	66.50 ^b^	61.15 ^b^	76.39 ^a^	75.42 ^a^	1.789	0.001
GSH, mg/gprot	129.18 ^a^	78.28 ^b^	79.00 ^b^	99.16 ^ab^	7.272	<0.001
MDA, nmol/mgprot	5.60	5.61	5.18	5.14	0.202	0.787
GSH-Px, U/mgprot	21.33 ^ab^	18.43 ^b^	24.45 ^a^	18.95 ^b^	0.816	0.043

^1^ CON: basal diet; MC: naturally mycotoxin-contaminated corn substituting for 73% of corn in CON; MA: MC with 1 g/kg mycotoxin adsorbent; MD: MC with 1 g/kg mycotoxin degradation agent. MDA: malondialdehyde; GSH: reduced glutathione; FRAR: ferric reducing antioxidant potential; CAT: catalase; T-SOD: superoxide dismutase; GSH-Px: glutathione peroxide; SEM: standard error of the mean. ^a,b^ Values in the same row with different superscripts were significantly different (*p* < 0.05).

**Table 7:** The efficacy of two mycotoxin detoxifications on serum biochemistry of laying hens fed diet naturally contaminated with low-level mycotoxins.

Items ^1^	CON	MC	MA	MD	SEM	*p* Value
ALT, U/L	7.52 ^b^	10.48 ^a^	7.37 ^b^	5.61 ^b^	0.534	0.003
AST, U/L	20.69	21.62	24.70	23.36	0.844	0.365

^1^ CON: basal diet; MC: naturally mycotoxin-contaminated corn substituting for 73% of corn in CON; MA: MC with 1 g/kg mycotoxin adsorbent; MD: MC with 1 g/kg mycotoxin degradation agent. ALT: alanine aminotransferase; AST: aspartate aminotransferase; SEM: standard error of the mean. ^a,b^ Values in the same row with different superscripts were significantly different (*p* < 0.05).

## Data Availability

The original contributions presented in this study are included in the article. Further inquiries can be directed to the corresponding author.

## Abbreviations

The following abbreviations are used in this manuscript:

AFB_1_	Aflatoxin B_1_
DON	Deoxynivalenol
ZEA	Zearalenone
MDA	Malondialdehyde
GSH	Reduced glutathione
FRAP	Ferric reducing antioxidant potential
CAT	Catalase
T-SOD	Total superoxide dismutase
GSH-Px	Glutathione peroxidase
AST	Aspartate aminotransferase
ALT	Alanine aminotransferase
H&E	Hematoxylin and eosin

## References

[1] Ochieng P.E., Kemboi D.C., Okoth S., De Baere S., Cavalier E., Kang’ethe E., Doupovec B., Gathumbi J., Scippo M.L., Antonissen G. (2025). Aflatoxins and fumonisins co-contamination effects on laying hens and use of mycotoxin detoxifiers as a mitigation strategy. Mycotoxin Res..

[2] Liu L., Xie M., Wei D. (2022). Biological Detoxification of Mycotoxins: Current Status and Future Advances. Int. J. Mol. Sci..

[3] Liu T., Ma Q., Zhao L., Jia R., Zhang J., Ji C., Wang X. (2016). Protective Effects of Sporoderm-Broken Spores of Ganderma lucidum on Growth Performance, Antioxidant Capacity and Immune Function of Broiler Chickens Exposed to Low Level of Aflatoxin B_1_. Toxins.

[4] Lucke A., Doupovec B., Paulsen P., Zebeli Q., Böhm J. (2017). Effects of low to moderate levels of deoxynivalenol on feed and water intake, weight gain, and slaughtering traits of broiler chickens. Mycotoxin Res..

[5] Men Y., Zhao Y., Zhang P., Zhang H., Gao Y., Liu J., Feng Y., Li L., Shen W., Sun Z. (2019). Gestational exposure to low-dose zearalenone disrupting offspring spermatogenesis might be through epigenetic modifications. Basic Clin. Pharmacol. Toxicol..

[6] Zhao L., Feng Y., Wei J.T., Zhu M.X., Zhang L., Zhang J.C., Karrow N.A., Han Y.M., Wu Y.Y., Guo Y.M. (2021). Mitigation Effects of Bentonite and Yeast Cell Wall Binders on AFB_1_, DON, and OTA Induced Changes in Laying Hen Performance, Egg Quality, and Health. Toxins.

[7] Zhu F., Zhu L., Xu J., Wang Y., Wang Y. (2023). Effects of moldy corn on the performance, antioxidant capacity, immune function, metabolism and residues of mycotoxins in eggs, muscle, and edible viscera of laying hens. Poult. Sci..

[8] Calado T., Venâncio A., Abrunhosa L. (2014). Irradiation for Mold and Mycotoxin Control: A Review. Compr. Rev. Food Sci. Food Saf..

[9] Olopade B.K., Oranusi S.U., Nwinyi O.C., Lawal I.A., Gbashi S., Njobeh P.B. (2019). Decontamination of T-2 Toxin in Maize by Modified Montmorillonite Clay. Toxins.

[10] Demirci A.S., Tirpanci Sivri G., Tunc M., Mutlu S. (2023). Detoxification of unshelled hazelnut artificially contaminated with aflatoxins by gaseous ozone. Food Meas..

[11] Liu M., Zhang X., Luan H., Zhang Y., Xu W., Feng W., Song P. (2024). Bioenzymatic detoxification of mycotoxins. Front. Microbiol..

[12] Elliott C.T., Connolly L., Kolawole O. (2020). Potential adverse effects on animal health and performance caused by the addition of mineral adsorbents to feeds to reduce mycotoxin exposure. Mycotoxin Res..

[13] Ruan M.L., Wang J., Xia Z.Y., Li X.W., Zhang B., Wang G.L., Wu Y.Y., Han Y., Deng J., Sun L.H. (2023). An integrated mycotoxin-mitigating agent can effectively mitigate the combined toxicity of AFB_1_, DON and OTA on the production performance, liver and oviduct health in broiler breeder hens. Food Chem. Toxicol..

[14] Kihal A., Rodríguez-Prado M., Calsamiglia S. (2022). The efficacy of mycotoxin binders to control mycotoxins in feeds and the potential risk of interactions with nutrient: A review. J. Anim. Sci..

[15] Guo H., Wang P., Liu C., Chang J., Yin Q., Wang L., Jin S., Zhu Q., Lu F. (2023). Compound mycotoxin detoxifier alleviating aflatoxin B_1_ toxic effects on broiler growth performance, organ damage and gut microbiota. Poult. Sci..

[16] National Research Council (1994). Nutrient Requirements of Poultry: Ninth Revised Edition, 1994. Washington, DC: The National Academies Press..

[17] (2017). Hygienical Standard for Feeds.

[18] Food and Drug Administration (2010). Guidance for Industry and FDA: Advisory Levels for Deoxynivalenol (DON) in Finished Wheat Products for Human Consumption and Grains and Grain By-Products Used for Animal Feed. https://www.fda.gov/regulatory-information/search-fda-guidance-documents/guidance-industry-and-fda-advisory-levels-deoxynivalenol-don-finished-wheat-products-human.

[19] (2006). The Commission of the European Communities. Commission recommendation of 17 August 2006 on the presence of deoxynivalenol, zearalenone, ochratoxin A, T-2 and HT-2 and fumonisins in products intended for animal feeding. Off. J. Eur. Union.

[20] Alharthi A.S., Al Sulaiman A.R., Aljumaah R.S., Alabdullatif A.A., Ferronato G., Alqhtani A.H., Abudabos A.M. (2022). The efficacy of bentonite and zeolite in reducing aflatoxin B_1_ toxicity on production performance and intestinal and hepatic health of broiler chickens. Ital. J. Anim. Sci..

[21] Bari M.S., Cohen-Barnhouse A.M., Campbell D.L.M. (2020). Early rearing enrichments influenced nest use and egg quality in free-range laying hens. Animal.

[22] Chen J., Zhou Z.J., Liu N.Z., Xie Q.X., Xue H.Y., Gu W., Wang C.F., Yin J.X. (2017). Effect of complex mycotoxin detoxification on performance, egg quality and serum biochemical indices in Hyland Brown laying hens. Feed. Ind. Mag..

[23] Qiu Q., Zhan Z., Zhou Y., Zhang W., Gu L., Wang Q., He J., Liang Y., Zhou W., Li Y. (2024). Effects of Yeast Culture on Laying Performance, Antioxidant Properties, Intestinal Morphology, and Intestinal Flora of Laying Hens. Antioxidants.

[24] Mavrommatis A., Giamouri E., Tavrizelou S., Zacharioudaki M., Danezis G., Simitzis P.E., Zoidis E., Tsiplakou E., Pappas A.C., Georgiou C.A. (2021). Impact of Mycotoxins on Animals’ Oxidative Status. Antioxidants.

[25] Bai K., Feng C., Jiang L., Zhang L., Zhang J., Zhang L., Wang T. (2018). Dietary effects of Bacillus subtilis fmbj on growth performance, small intestinal morphology, and its antioxidant capacity of broilers. Poult. Sci..

[26] Qiu Z., Wang H., Li G., Liu Y., Wang X., Yang J., Wang X., He D. (2024). Lactobacillus salivarius Ameliorates AFB_1_-induced hepatotoxicity via PINK1/Parkin-mediated mitophagy in Geese. Ecotoxicol. Environ. Saf..

[27] Skiepko N., Przybylska-Gornowicz B., Gajęcka M., Gajęcki M., Lewczuk B. (2020). Effects of Deoxynivalenol and Zearalenone on the Histology and Ultrastructure of Pig Liver. Toxins.

[28] Xu J., Li S., Jiang L., Gao X., Liu W., Zhu X., Huang W., Zhao H., Wei Z., Wang K. (2021). Baicalin protects against zearalenone-induced chicks liver and kidney injury by inhibiting expression of oxidative stress, inflammatory cytokines and caspase signaling pathway. Int. Immunopharmacol..

[29] Hasuda A.L., Person E., Khoshal A.K., Bruel S., Puel S., Oswald I.P., Bracarense A.P.F.R.L., Pinton P. (2022). Deoxynivalenol induces apoptosis and inflammation in the liver: Analysis using precision-cut liver slices. Food Chem. Toxicol..

[30] Chen Z., Chen R., Ma X., Wu W., Huang Q., Ye W., Wu C., Yao B., Xu J., Qian L. (2024). A Multi-Enzyme Complex That Mitigates Hepatotoxicity, Improves Egg Production and Quality, and Enhances Gut and Liver Health in Laying Hens Exposed to Trace Aflatoxin B_1_. Toxins.

[31] Zabiulla I., Malathi V., Swamy H.V.L.N., Naik J., Pineda L., Han Y. (2021). The Efficacy of a Smectite-Based Mycotoxin Binder in Reducing Aflatoxin B_1_ Toxicity on Performance, Health and Histopathology of Broiler Chickens. Toxins.

[32] Ralla K., Sohling U., Riechers D., Kasper C., Ruf F., Scheper T. (2010). Adsorption and separation of proteins by a smectitic clay mineral. Bioprocess Biosyst. Eng..

[33] Barrientos-Velazquez A.L., Arteaga S., Dixon J.B., Denget Y.J. (2016). The effects of pH, pepsin, exchange cation, and vitamins on aflatoxin adsorption on smectite in simulated gastric fluids. Appl. Clay Sci..

[34] Pimpukdee K., Kubena L.F., Bailey C.A., Huebner H.J., Afriyie-Gyawu E., Phillips T.D. (2004). Aflatoxin-induced toxicity and depletion of hepatic vitamin A in young broiler chicks: Protection of chicks in the presence of low levels of NovaSil PLUS in the diet. Poult. Sci..

[35] Kihal A., Marquès C., Rodríguez-Prado M., Jose-Cunilleras E., Calsamiglia S. (2022). Effect of Diet Supplementation with the Mycotoxin Binder Montmorillonite on the Bioavailability of Vitamins in Dairy Cows. Toxins.

[36] Jahanian R., Ashnagar M. (2015). Effect of dietary supplementation of mannan-oligosaccharides on performance, blood metabolites, ileal nutrient digestibility, and gut microflora in Escherichia coli-challenged laying hens. Poult. Sci..

[37] Rizzi L., Simioli M., Roncada P., Zaghini A. (2003). Aflatoxin B_1_ and clinoptilolite in feed for laying hens: Effects on egg quality, mycotoxin residues in livers, and hepatic mixed-function oxygenase activities. J. Food Prot..

[38] Wang Q., Zhan X., Wang B., Wang F., Zhou Y., Xu S., Li X., Tang L., Jin Q., Li W. (2022). Modified Montmorillonite Improved Growth Performance of Broilers by Modulating Intestinal Microbiota and Enhancing Intestinal Barriers, Anti-Inflammatory Response, and Antioxidative Capacity. Antioxidants.

[39] Awais M.M., Mehtab U., Anwar M.I., Hameed M.R., Akhtar M., Raza A., Aisha R., Muhammad F., Saleemi M.K., Fayyaz A. (2022). Mitigation potential of individual and combined dietary supplementation of local Bentonite Clay and Distillery Sludge against Ochratoxin-A induced toxicity in broilers. BMC Vet. Res..

[40] Mashkoor J., Al-Saeed F.A., Guangbin Z., Alsayeqh A.F., Gul S.T., Hussain R., Ahmad L., Mustafa R., Farooq U., Khan A. (2023). Oxidative stress and toxicity produced by arsenic and chromium in broiler chicks and application of vitamin E and bentonite as ameliorating agents. Front. Vet. Sci..

[41] Murugesan G.R., Ledoux D.R., Naehrer K., Berthiller F., Applegate T.J., Grenier B., Phillips T.D., Schatzmayr G. (2015). Prevalence and effects of mycotoxins on poultry health and performance, and recent development in mycotoxin counteracting strategies. Poult. Sci..

[42] Ndiaye S., Zhang M., Fall M., Ayessou N.M., Zhang Q., Li P. (2022). Current Review of Mycotoxin Biodegradation and Bioadsorption: Microorganisms, Mechanisms, and Main Important Applications. Toxins.

